# Supplementary material for: Combined Transcranial Direct Current Stimulation and Functional Electrical Stimulation for Upper Limbs in Individuals with Stroke: A Systematic Review
Source: Neurol Int. 2025 Jun 9;17(6):89. doi: 10.3390/neurolint17060089 (PMC12195863; doi:10.3390/neurolint17060089)
Supplement: Supplementary file 1 [file neurolint-17-00089-s001.zip › Supplemental material_search strategy.pdf]

Search Strategy

PUBMED: 176 articles

((“noninvasive brain stimulation” OR “non-invasive brain stimulation” OR “NIBS”) OR (“transcranial direct current stimulation”[MeSH Terms] OR “transcranial direct current stimulation” OR “tDCS” OR “transcranial electrical stimulation”)) AND (“electric stimulation therapy”[MeSH Terms] OR “Electrical stimulation” OR “Functional electrical stimulation” OR “Neuromuscular electrical stimulation” OR “FES” OR “NMES”) AND ((“paresis”[MeSH Terms] OR “hemiparesia” OR “hemiparesis” OR “hemipareses”) OR (“Stroke” OR “Stroke”[Mesh Terms] OR “Cerebrovascular accident” OR “CVA”)) AND (“upper limb” OR hand OR arm OR “upper extremity”[Mesh]) NOT ("review" OR "protocol*")

SCOPUS: 85 articles

TITLE-ABS-KEY(“noninvasive brain stimulation” OR “non-invasive brain stimulation” OR “NIBS” OR “transcranial direct current stimulation” OR “transcranial direct current stimulation” OR “tDCS”) AND TITLE-ABS-KEY(“electric stimulation therapy” OR “Electrical stimulation” OR “Functional electrical stimulation” OR “Neuromuscular electrical stimulation” OR “FES” OR “NMES”) AND TITLE-ABS-KEY(“paresis” OR “hemiparesia” OR “hemiparesis” OR “hemipareses” OR “Stroke” OR “Stroke” OR “Cerebrovascular accident” OR “CVA”) AND TITLE-ABS-KEY(“upper limb” OR hand OR arm OR “upper extremity”) AND NOT TITLE("review" OR "protocol")

COCHRANE: 289 trials

ID	Search	Hits
#1	(noninvasive brain stimulation OR non-invasive brain stimulation OR NIBS):ti,ab,kw (Word variations have been searched)	2979
#2	MeSH descriptor: [Transcranial Direct Current Stimulation] explode all trees	2109
#3	transcranial direct curent stimulation OR tDCS OR transcranial electrical stimulation	6984
#4	#1 OR #2 OR #3	9446
#5	MeSH descriptor: [Electric Stimulation Therapy] explode all trees	11565
#6	Electrical stimulation OR Functional electrical stimulation OR Neuromuscular electrical stimulation OR FES OR NMES	14729
#7	#5 OR #6	23265
#8	MeSH descriptor: [Paresis] explode all trees	1141
#9	hemiparesia OR hemiparesis OR hemipareses	1901
#10	MeSH descriptor: [Stroke] explode all trees	18294
#11	cerebrovascular accident OR CVA	20085
#12	#8 OR #9 OR #10 OR #11	35043
#13	MeSH descriptor: [Upper Extremity] explode all trees	10905
#14	upper limb OR hand OR arm OR upper extremity	198534
#15	#13 OR #14	202122
#16	#4 AND #7 AND #12 AND #14	289

Web Of Science: 43 documents

((((TS=("noninvasive brain stimulation" OR "non-invasive brain stimulation" OR "NIBS" OR "transcranial direct current stimulation" OR "tDCS" OR "transcranial electrical stimulation")) AND TS=("electric stimulation therapy" OR "electrical stimulation" OR "functional electrical stimulation" OR "neuromuscular electrical stimulation" OR "FES" OR "NMES")) AND TS=("paresis" OR "hemiparesis" OR "stroke" OR "cerebrovascular accident" OR "CVA")) AND TS=("upper limb" OR hand OR arm OR "upper extremity")) NOT TS=("review" OR "protocol")

EBSCO (CINAHL & SPORTDISCUS): 5 articles

AB ((“noninvasive brain stimulation” OR “non-invasive brain stimulation” OR “NIBS” OR “transcranial direct current stimulation” OR “transcranial direct current stimulation” OR “tDCS” OR “transcranial electrical stimulation”)) AND AB ((“electric stimulation therapy” OR “Electrical stimulation” OR “Functional electrical stimulation” OR “Neuromuscular electrical stimulation” OR “FES” OR “NMES”)) AND AB ((“paresis” OR “hemiparesia” OR “hemiparesis” OR “hemipareses” OR “Stroke” OR “Stroke” OR “Cerebrovascular accident” OR “CVA”)) AND AB ((“upper limb” OR hand OR arm OR “upper extremity”)) NOT TI (("review" OR "protocol"))
